# Calcitriol-enhanced autophagy in gingival epithelium attenuates periodontal inflammation in rats with type 2 diabetes mellitus

**DOI:** 10.3389/fendo.2022.1051374

**Published:** 2023-01-10

**Authors:** Yanan Wang, Maoting Huang, Wanlin Xu, Fulong Li, Chunliang Ma, Xiaolin Tang

**Affiliations:** Department of Periodontology, School and Hospital of Stomatology, China Medical University, Liaoning Provincial Key Laboratory of Oral Diseases, Shenyang, China

**Keywords:** type 2 diabetes mellitus, *Porphyromonas gingivalis*, calcitriol, LL-37, autophagy, rats model

## Abstract

Type 2 diabetes mellitus (T2DM)-associated periodontitis is a common disease with high prevalence, associated with persistent infection and complicated manifestations. Calcitriol (1 alpha, 25-dihydroxyvitamin D3, 1,25D) is the active form of vitamin D that plays a protective role in immune regulation, bone metabolism, and inflammatory response. In this study, we constructed a T2DM model in rats by combining a high-fat diet with low-dose streptozotocin. The periodontitis model in rats was developed by ligation and *Porphyromonas gingivalis* (ATCC 33277) inoculation. Rats were randomly divided into five groups: non-diabetic blank, diabetic blank, diabetes with calcitriol treatment, diabetes with 3-methyladenine (3-MA) treatment, or diabetes with calcitriol and 3-MA treatment. The diabetic rats exhibited an intense inflammatory response and decreased autophagy compared with the non-diabetic rats. Intraperitoneal injection of calcitriol and autophagy inhibitor (3-MA) allowed us to explore the effect of calcitriol on inflammation in the gingival epithelium and the role of autophagy in this process. Treatment with calcitriol resulted in the decreased expression of NFκB-p65, p62/SQSTM1 and inflammatory response and increased expression of LC3-II/LC3-I. Application of 3-MA significantly suppressed autophagy, which was apparently retrieved by calcitriol. Antibacterial peptide (LL-37) is the only antimicrobial peptide in the cathelicidin family that is found in the human body, and it exhibits a broad spectrum of antibacterial activity and regulates the immune system. In the present study, our findings indicated that calcitriol-enhanced autophagy may attenuated periodontitis and the decrease of LL-37 was rescued by calcitriol treatment in the gingival epithelial cells of T2DM rats. Our study provides evidence for the application of calcitriol as an adjunctive treatment for T2DM-associated periodontitis.

## Introduction

Diabetes mellitus is a metabolic disorder characterized by chronic hyperglycemia. It has the third highest health risk of disability and mortality after tumors and cardiovascular disease. Periodontitis is one of the most frequent complications of diabetes, especially type 2 diabetes mellitus (T2DM) (1). The clinical manifestations of periodontitis are periodontal inflammation and attachment loss (AL), which subsequently exacerbate alveolar bone resorption and tooth loss. The Global Burden of Diseases, Injuries, and Risk Factors Study 2017 (GBD 2017) estimated that severe periodontitis affects 11% of the global population (2, 3). The prevalence of periodontitis in diabetics is significantly higher than that in non-diabetics (17.3% vs 9%) (4). There is evidence that those with moderate-to-severe periodontitis have a significantly increased risk of incident T2DM (5). The impaired immune function of people with diabetes leads to impaired resistance against periodontal infection, which exacerbates alveolar bone loss and impairs tissue healing (6). Moreover, severe periodontitis aggravates insulin resistance, making achieving clinical stability in diabetes challenging (7). In summary, diabetics have intense clinical manifestations and poor therapeutic responsiveness. Therefore, it is essential to improve the clinical diagnosis and treatment efficacy of diabetes mellitus-associated periodontitis.

Vitamin D is an essential regulating hormone in the human body. It is converted into its active form, calcitriol (1 alpha, 25-dihydroxyvitamin D3, 1,25D), and circulates through the blood to target organs (8). Based on the effects of vitamin D on immune regulation, bone metabolism, and inflammatory response, the relationship between calcitriol and diabetes mellitus-associated periodontitis has been explored in several recent studies. Vitamin D deficiency increases the risk of periodontitis and the progressive destruction of periodontal tissues (9). Our previous and related studies reported that calcitriol can alleviate periodontal inflammation by inhibiting endogenous expression of IL-8 in human periodontal ligament cells (hPDLCs) and the expression of IL-8/IL-6 in hPDLCs induced by *Phorphyromonas gingivalis* lipopolysaccharide (10). Wang et al. (11) found that calcitriol attenuated experimental periodontitis through downregulation of TLR4 and JAK1/STAT3 signaling in diabetic mice. In human oral keratinocytes and a mouse model of T2DM, PTPN2 contributed to a decrease in periodontal inflammation *via* protein substrate dephosphorylation in the JAK1/STAT3 signaling pathway after calcitriol treatment (12). However, it remains unclear whether there are other mechanism involved in vitamin D-induced alleviation of periodontitis in T2DM. Thorough investigation of calcitriol and its involvement in inhibiting inflammation may help improve the treatment of diabetes mellitus-associated periodontitis.

Autophagy is a physiological process by which cells phagocytize their organelles or cytoplasm and degrade the cargo in lysosomes (13). It is a self-protective cellular catabolic pathway that is essential for cellular homeostasis, immune defense, and stress response (14). Autophagy plays an important role in the development of periodontitis. Increased expression of autophagy-related protein was found in PDLCs from individuals with periodontitis compared with healthy individuals (15). Park et al. (16) found that THP-1-derived macrophages eliminated *P. gingivalis* and restricted excessive inflammatory response by activating an autophagic response. Moreover, increased autophagy induced osteoclastogenesis and stimulated osteoclast-mediated bone resorption. However, the role of autophagy in periodontitis and its related mechanisms remain unclear.

Vitamin D can regulate the inflammatory response by promoting autophagy in various tissues. A study found that active vitamin D activated chondrocyte autophagy to reduce osteoarthritis (17). Wei et al. (18) discovered that vitamin D protected against myocardial damage, inflammation, and apoptosis by promoting autophagy. In addition, vitamin D has potential renoprotective effects in diabetic nephropathy *via* downregulation of mTOR gene expression, stimulation of autophagy, and antioxidant, anti-inflammatory, and hypotensive effects (19). In our previous study, we found that calcitriol significantly decreased the number of live *P. gingivalis* internalized into epithelial cells, monocytes, and macrophages by promoting autophagy (20, 21). Human cationic antimicrobial peptide 18 (hCAP18) is the only antimicrobial peptide in the cathelicidin family found in the human body. LL-37 is expressed in salivary glands, oral mucosa, and oral immune cells (22). We have previously found that LL-37 reduced the quantity of live *P. gingivalis* internalized into HaCaT cells by promoting autophagy, and a potential molecular pathway of LL-37-induced autophagy was indicated (23). However, the role of autophagy in the regulation of periodontal inflammatory response treated with vitamin D in diabetes mellitus-associated periodontitis remains unclear.

Our previous study found that the calcitriol treatment may inhibit the number of live *P. gingivalis* in KB cells and U-937 cells by promoting autophagy. But the effect of calcitriol on the inflammatory responses of gingival tissues with T2DM-associated periodontitis has been rarely studied, and the role of autophagy during the inflammatory process is still unclear. In this study, the model of experimental T2DM with periodontitis in rats was employed to investigate the effect of calcitriol treatment on the regulation of inflammation in the gingival tissues, especially in the epithelial cells. In addition, we also explored the possible roles of autophagy and LL-37 during the process so as to provide further evidence for relevant mechanisms.

## Materials and methods

### Animals, bacterial strains, and grouping

Twenty male Sprague-Dawley rats (7 weeks old) were purchased from Beijing Vital River (Beijing, China). All rats were given free access to food and tap water and were caged on an auto-cycling 12-h light and 12-h dark cycle under specific pathogen-free conditions. The *P. gingivalis* ATCC 33277 strain, which is the most common putative pathogen involved in periodontitis, was originally obtained from the American Tissue Culture Collection (Maryland, USA) and stored at the Department of Oral Biology at China Medical University.

Rats were randomly divided into five groups: non-diabetic blank, diabetic blank, diabetes with calcitriol treatment, diabetes with 3-methyladenine (3-MA) treatment, or diabetes with calcitriol and 3-MA treatment. All experimental procedures were approved by the Animal Ethics Committee of China Medical University (KT2019040).

### High-fat diet/streptozotocin-induced experimental type 2 diabetes model in rats

The rats in the diabetic groups consumed high-fat diet (HFD, HD001, 60% calories from fat) for 30 days. Then, they were fasted overnight before receiving intraperitoneal injection of a low dose of streptozotocin (STZ) (35 mg/kg), according to Srinivasan et al. (24) Three days after injection, random blood glucose (RBG) measurement was performed. The model was considered successful if the RBG was not less than 16.7 mmol/L.

### Ligation/*P. gingivalis*-induced experimental periodontitis model in rats

The *P. gingivalis* ATCC 33277 strain was maintained anaerobically at 37°C on brain-heart infusion (BHI) solid medium (containing 5% sterilized and defibrinated sheep blood, 0.5% hemin, and 0.1% vitamin K). Then *P. gingivalis* was cultured in a liquid BHI medium for 16–18 h. After the type 2 diabetes model was established, 5-0 silk ligatures were ligated firmly and sub-gingivally around the left maxillary first molar to facilitate bacterial colonization; the right side was the self-control. Then, they were infected with 10^9^ colony-forming units of live bacteria inoculated into the gingival sulci by a syringe three times at 2 days.

### Calcitriol treatment and autophagy blocking

After the rat model was established, intraperitoneal injection of blank solvent (propylene glycol:water:ethyl glycol = 60:30:10), calcitriol solvent (2 µg/kg, Sigma, St. Louis, MO, USA), 3-MA solvent (15 mg/kg, Selleck, Texas, USA), and calcitriol + 3-MA solvent was performed for 1 week. All rats were sacrificed for subsequent experiments.

### Micro-computed tomography and histological analysis

After stripping the attached gingival tissue, all maxillary teeth and alveolar bone fixed in 4% paraformaldehyde were subjected to scanning by a micro-computed tomography system.

The gingival samples were fixed with 4% paraformaldehyde for 24–48 hours, embedded, and sliced. The slices were prepared for hematoxylin and eosin (H&E) staining.

### Immunohistochemical analysis

After being deparaffinized, rehydrated, and antigen retrieved, the sample slices were quenched with endogenous peroxidase and blocked. Then, sample slices were incubated with primary antibody overnight, including nuclear transcription factor-kappa B p65 (NFκB-p65, Immunoway, China, YT3107,1:200), microtubule-associated proteins 1A/1B light chain 3B (LC3B, Proteintech, China, 14600-1, 1:500), and antibacterial peptide LL-37 (LL-37, Cohesion Biosciences, England, CQA1065, 1:200). On the next day, the processes of secondary antibody binding, DAB, hematoxylin re-staining, dehydration, transparency, and sealing were performed to complete immunohistochemistry.

### Western blot analysis

The gingival tissue was lysed with a RIPA lysis buffer supplemented with 1 mM PMSF. Then, samples were centrifuged at 12,000 rpm at 4 °C for 15 min in three freezing-melting cycles. Equal amounts of protein samples were separated by SDS-polyacrylamide gel electrophoresis and transferred onto a PVDF membrane by electro-blotting. The membranes were blocked with 5% BSA dissolved in Tris-buffered saline containing Tween (TBST), then incubated with primary antibodies at 4 °C overnight, including LC3B (1:1000), p62/SQSTM1 (GeneTex, America, GTX636328, 1:2000), NFκB-p65 (1:1000), GAPDH (Proteintech, China, 10494-1, 1:2000) and LL-37 (1:1000) at 4 °C. Membranes were incubated with a secondary antibody (Abbkine, America, A23220, 1:1000) in the dark for 1 h at 37 °C. Images were obtained using the Infrared Fluorescence Scanning Imaging System (Odyssey CLx, LI-COR, USA).

### Statistical analysis

All the experiments were conducted in triplicate. The data are presented as mean ± standard deviation (SD). The differences between two groups were assessed by unpaired *t* test, and differences among three or more groups were analyzed by one-way analysis of variance (ANOVA) using SPSS 22.0 (SPSS Inc., Chicago, IL, USA). *P* < 0.05 was considered statistically significant.

## Results

### Establishment of the experimental type 2 diabetes model in rats

**Figure 1:**
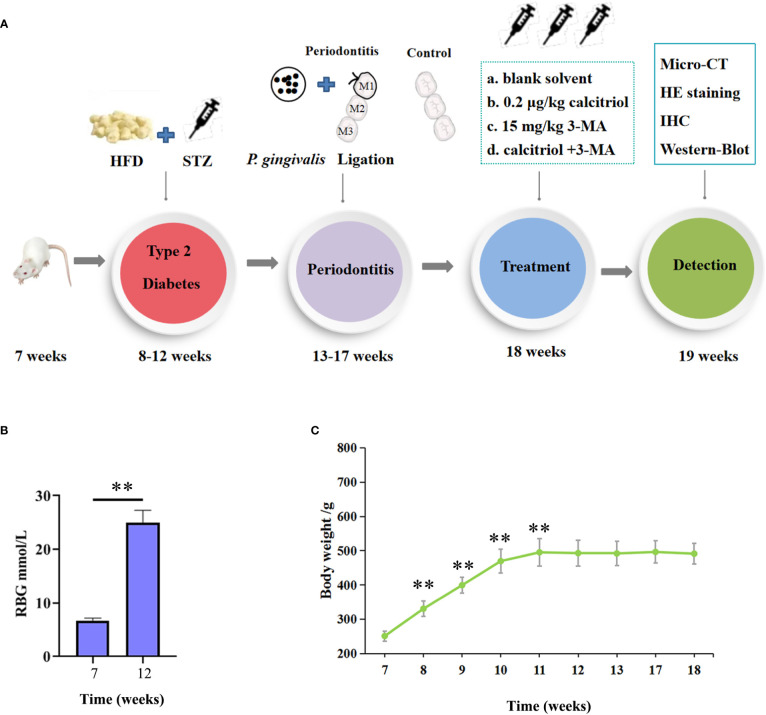
Establishment of the experimental type 2 diabetic model in rats. **(A)** The flow chart of the experimental design. HFD, High-Fat Diet; STZ, streptozotocin (35 mg/kg); HE, hematoxylin and eosin; IHC, immunohistochemical. **(B)** The random blood glucose (RBG) of the rats were measured. **(C)** Body weight of the diabetic rats were measured. The data were presented as the mean ± SD (n=4/group, **P <0.01, compared with 7 week).

As shown in the flow chart of operating processes (**Figure 1A**), we combined a high-fat diet (HFD) and low dose of STZ to develop an experimental type 2 diabetes model in rats. After HFD and injection of STZ, the random blood glucose (RBG) of rats in our study ranged from 20.9 mmol/L to 28.2 mmol/L (**Figure 1B**). The RBG was not less than 16.7 mmol/L in diabetic rats, which indicated a successful model. The body weight of all rats was measured for 8 weeks. Body weight gradually increased for 4 weeks, and the rats exhibited a stable or slight decrease in body weight after intraperitoneal injection of STZ (**Figure 1C**). The changes in RBG and body weight in this study indicated the establishment of the experimental type 2 diabetes model in the rats.

### Establishment of the experimental periodontitis model in rats

**Figure 2:**
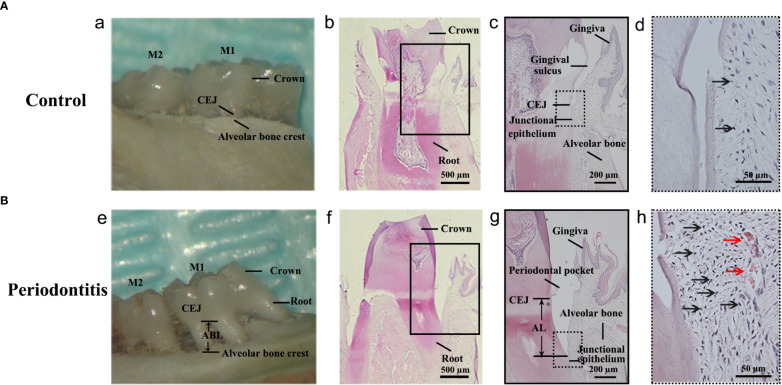
Establishment of the experimental periodontitis model in rats. **(A, B)** The figures presented the gross specimens and sections obtained individually from the control group and the periodontitis group. (a, e): As shown in the gross specimen, the distance from the cemento-enamel junction (CEJ) to the alveolar bone crest in the periodontitis group was remarkably longer than that in the control group, that is alveolar bone resorption occurred. (b, c): As shown in the section from the control group with hematoxylin and eosin (HE) staining, the junctional epithelium is located at the CEJ site. (f, g): The section from the periodontitis group shows that the junctional epithelium was located on the root, that is attachment loss (AL) occurred. (d, h): Compared with that from the control group, more inflammatory cells and more severe vasodilatation was observed in the gingival tissue from the periodontitis group. The black arrows in (d, h) indicates the inflammatory cell (especially neutrophile) infiltration. The red arrows in (h) indicates vasodilatation. M1, the maxillary first molar; M2, the maxillary second molar.

The maxillary bone samples were obtained after sacrifice. The inflammatory responses were observed in the gross specimen and *via* H&E staining of samples obtained individually from the control group and the periodontitis group (**Figure 2A, B**). As shown in the gross specimen (**Figure 2A a; Figure 2B e**), the distance from the cemento-enamel junction (CEJ) to the alveolar bone crest in the periodontitis group was remarkably longer than in the control group, indicating that alveolar bone resorption had occurred. Compared with H&E staining of gingival tissue from the control group, the location of the junctional epithelium in the periodontitis group, from CEJ to root, indicated AL (**Figure 2A b, c; Figure 2B f, g**). Moreover, more inflammatory cells and more severe vasodilatation was observed in the gingival tissue from the periodontitis group than the control group (**Figure 2A d; Figure 2B h**). The occurrence of AL, inflammatory cell infiltration, and severe vasodilatation indicated the establishment of the experimental periodontitis model in rats.

### Diabetic rats with periodontitis showed more severe alveolar bone loss and intense gingival inflammatory response than non-diabetic rats

**Figure 3:**
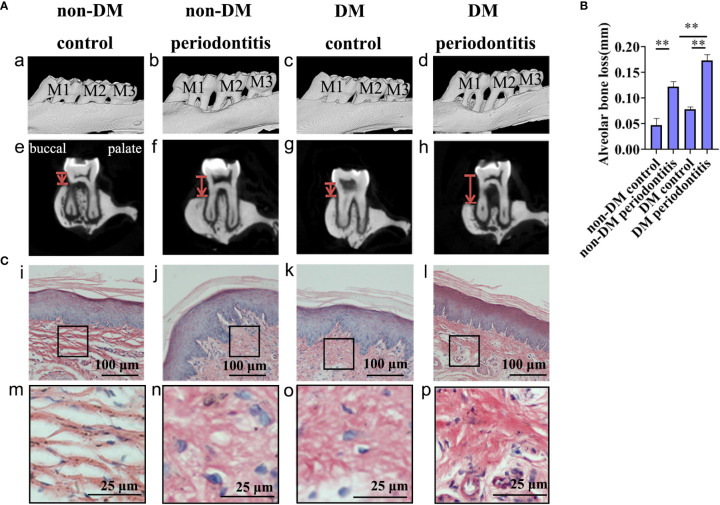
Diabetic rats with periodontitis showed more severe alveolar bone loss and intense gingival inflammatory response than non-diabetic rats. The figures present the micro-CT images and sections obtained individually from the non-DM control group, non-DM periodontitis group, DM control group and the DM periodontitis group. Non-DM control group, health control without type 2 diabetes mellitus (T2DM) and periodontitis; non-DM periodontitis group, ligation/*P. gingivalis-*induced periodontitis but without T2DM; DM control group, high-fat diet and low dose of streptozotocin-induced T2DM but without periodontitis; DM periodontitis group, combination periodontitis and T2DM. **(A)** Micro-computed tomography images of the maxillary first molars from (a-d). The distance between two red lines, cemento-enamel junction (CEJ) and the alveolar bone crest, represents the alveolar bone resorption (e-h). **(B)** Statistical analysis of the alveolar bone loss. Data are presented as mean ± SD (n=4) and are shown in the bar graphs, repeated 3 times (**P <0.01, compared with the control group or the non- DM group). **(C)** Hematoxylin and eosin staining of sections (i-p).

We performed Micro-CT of the maxillary bone and H&E staining of gingival tissues from non-DM control group (health control without T2DM and periodontitis), non-DM periodontitis group (ligation/*P. gingivalis-* induced periodontitis but without T2DM), DM control group (high-fat diet and low dose of streptozotocin-induced T2DM but without periodontitis), and DM periodontitis group (combination periodontitis and T2DM) to explore the different inflammatory response in four groups. The overall morphology, as a reconstructed 3D model, exhibited a significantly higher degree of bone loss in diabetic rats than in non-diabetic rats (**Figure 3A, B**). H&E staining revealed epithelial rete peg elongation, inflammatory cell infiltration, disordered arrangement of subepithelial fibers, and vascular reaction in rats with diabetes mellitus-associated periodontitis (**Figure 3C**). The above data indicated that the diabetic rats with periodontitis showed more alveolar bone loss and intense gingival inflammatory responses.

### Autophagy in gingival epithelium was attenuated in diabetic rats

**Figure 4:**
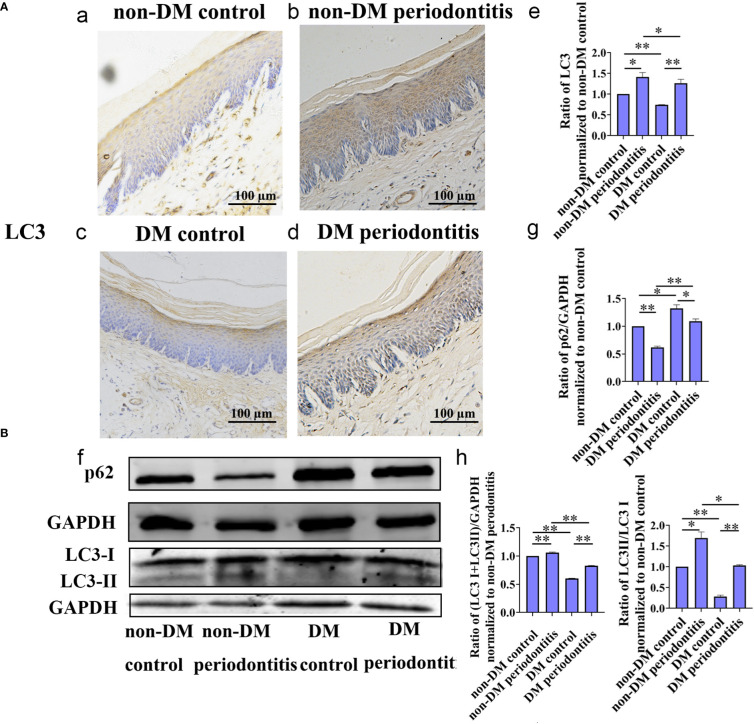
Autophagy in gingival epithelium was attenuated in diabetic rats. The expression of autophagy-related protein of gingival epithelium in non-DM control group, non-DM periodontitis group, DM control group and the DM periodontitis group were detected. Non-DM control group, health control without type 2 diabetes mellitus (T2DM) and periodontitis; non-DM periodontitis group, ligation/*P. gingivalis-*induced periodontitis but without T2DM; DM control group, high-fat diet and low dose of streptozotocin-induced T2DM but without periodontitis; DM periodontitis group, combination periodontitis and T2DM. **(A)** Immunohistochemical (IHC) staining and semi-quantitative analysis of the expression of LC3 in the gingival epithelium of the four groups (a-e). **(B)** Western Blot reveals the expression of LC3 and p62 in four groups (f-h). All data are presented as mean ± SD (n = 3) relative to non- DM control are shown in bar graphs (*P <0.05, **P <0.01).

Autophagy is usually assessed by detecting changes in LC3, LC3-II/LC3-I ratio and p62. The expression of LC3, LC3-II/LC3-I ratio and p62 in gingival epithelium of non-DM control group, non-DM periodontitis group, DM control group and DM periodontitis group were detected by immunohistochemical (IHC) and western blot. IHC staining showed the significantly decreasing expression of LC3 and western blot revealed the significantly decreasing expression of LC3 and LC3-II/LC3-I ratio and the increasing expression of p62 in the diabetic rats (**Figure 4**). The results indicated a marked decrease of autophagy in the diabetic gingival tissues, especially in the epithelial cells. Besides, autophagy was promoted in gingival tissues with periodontitis both in non-diabetic and diabetic rats.

### Calcitriol attenuated the gingival epithelium inflammatory responses in diabetic rats by promoting autophagy

**Figure 5:**
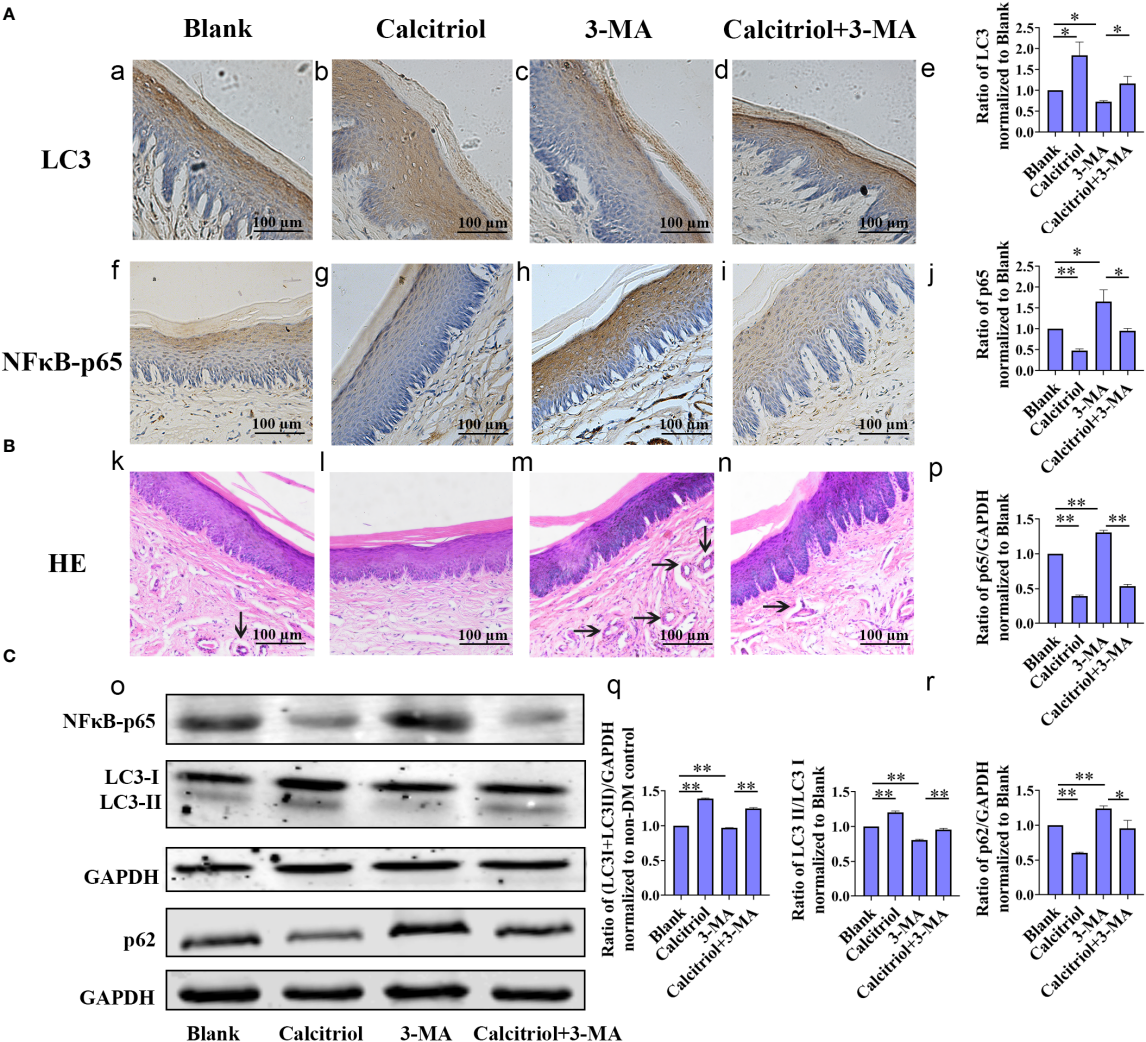
Calcitriol attenuated the gingival epithelium inflammatory response in diabetic rats by promoting autophagy. The diabetic rats with periodontitis were randomly divided into blank group (propylene glycol: water: ethyl glycol=60:30:10), calcitriol group (2 µg/kg), 3-MA group (15 mg/kg) and calcitriol + 3-MA group. They received intraperitoneal injections for 1 week. **(A)** The expression of LC3 (a-e) and NFκB-p65 (f-j) in the gingival epithelium of the four groups were detected by IHC staining. **(B)** HE staining showed the inflammatory response in the subepithelial fiber of the four groups (k-n). **(C)** The expression of NFκB-p65, LC3-II/LC3-I and p62 in the gingival epithelium of the four groups were detected by western-blot (o-r). Semi-quantitative analysis of IHC and western-blot were performed by gray density analysis. All data are presented as mean ± SD (n = 3) relative to blank group are shown in bar graphs (*P <0.05, **P <0.01).

To study the intrinsic mechanism underlying autophagy, the diabetic rats with periodontitis were randomly divided into blank group (propylene glycol: water: ethyl glycol=60:30:10), calcitriol group (2 µg/kg), 3-MA group (15 mg/kg) and calcitriol + 3-MA group. After intraperitoneal injections for 1 week, we harvested the gingival tissues following sacrifice of the rats. IHC staining was performed to evaluate the expression of inflammatory response-related proteins (NFκB-p65) and autophagy- related proteins (LC3) in the gingival epithelium (**Figure 5A**). Western blot was performed to detect the expression of LC3, LC3-II/LC3-I, p62 and NFκB-p65 (**Figure 5C**). HE staining was performed to observe the inflammatory response in the subepithelial fiber (**Figure 5B**). We observed that the expression of NFκB-p65 and p62 was alleviated by treatment with calcitriol (**Figure 5A f-j; Figure 5C o, p, r**). Furthermore, the expression of LC3 and LC3-II/LC3-I ratio were significantly elevated (**Figure 5A a-e; Figure 5C o, q**). Following intraperitoneal injection of 3-MA, the level of LC3 and LC3-II/LC3-I ratio were decreased (**Figure 5A a-e; Figure 5C o, q**), the expression of p62 and NFκB-p65 were increased (**Figure 5A f-j; Figure 5C o, p,r**), indicating marked suppression of autophagy. Autophagy suppression of 3-MA was apparently retrieved by calcitriol.

### Decrease of LL-37 in the gingival epithelial cells of T2DM rats, which was rescued by calcitriol treatment

**Figure 6:**
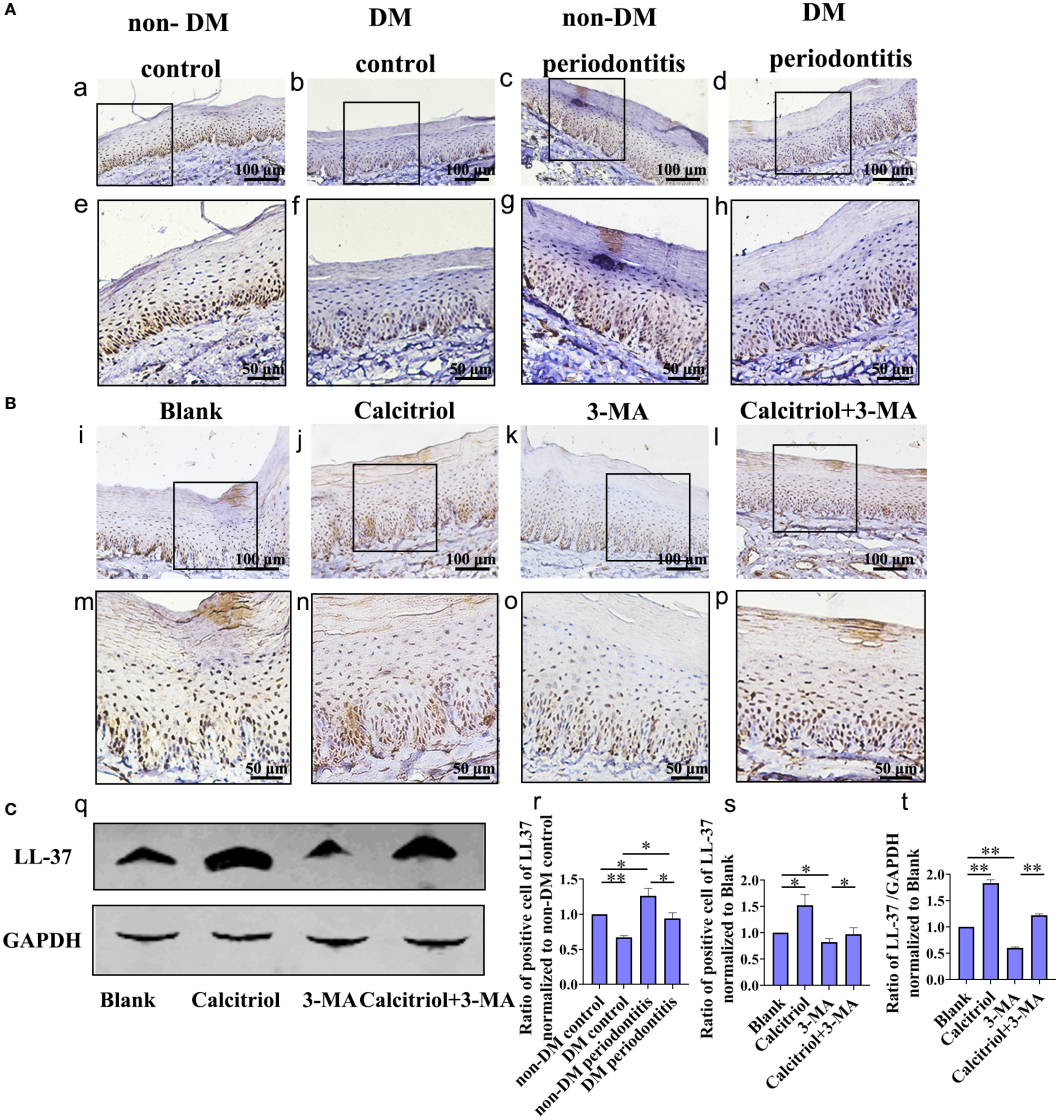
Decrease of LL-37 in the gingival epithelial cells of T2DM rats, which was rescued by calcitriol treatment. **(A)** IHC staining of the gingival epithelium obtained individually from the non-DM control group, non-DM periodontitis group, DM control group and the DM periodontitis group (a-h, r). Non-DM control group, health control without type 2 diabetes mellitus (T2DM) and periodontitis; non-DM periodontitis group, ligation/*P. gingivalis-*induced periodontitis but without T2DM; DM control group, high-fat diet and low dose of streptozotocin-induced T2DM but without periodontitis; DM periodontitis group, combination periodontitis and T2DM. **(B)** IHC staining of the LL-37 expression in the gingival epithelium of different groups (i-p, s). The diabetic rats with periodontitis were randomly divided into blank group (propylene glycol: water: ethyl glycol=60:30:10), calcitriol group (2 µg/kg), 3-MA group (15 mg/kg) and calcitriol + 3-MA group. **(C)** Western Blot reveals the expression of LL-37 in four groups (q) analysis of the LL-37 expression in the gingival epithelium (t). All data are presented as mean ± SD (n = 3) relative to non-DM and blank group are shown in bar graphs (*P <0.05, **P <0.01, compared with the blank group).

Based on our previous study on LL-37-induced autophagy, we further detected the expression of LL-37 in diabetics. IHC staining was performed to assess the expression of LL-37 in the gingival epithelium of non-DM control group, non-DM periodontitis group, DM control group and DM periodontitis group (**Figure 6A**). We observed a lower expression of LL-37 in the diabetic rats compared with non-diabetic rats and LL-37 was promoted in gingival tissues with periodontitis both in non-diabetic and diabetic rats (**Figure 6A a-h; Figure 6C r**).

We further explored the role of LL-37 in calcitriol-enhanced autophagy in diabetics rats with periodontitis, which randomly divided into blank group, calcitriol group, 3-MA group and calcitriol + 3-MA group (**Figure 6B, C**). We observed that the expression of LL-37 was increased by treatment with calcitriol (**Figure 6B j, n; Figure 6C q, s, t**). In contrast, LL-37 expression decreased following intraperitoneal injection of 3-MA (**Figure 6B k, o; Figure 6C q, s, t**). LL-37-mediated suppression of 3-MA was apparently retrieved by calcitriol treatment (**Figure 6B l, p; Figure 6C q, s, t**).

## Discussion

Autophagy plays an important role in maintaining general homeostasis, but the specific function and mechanism of autophagy in T2DM-associated periodontitis is not clear. In our study, we employed rats with T2DM and periodontitis to explore the effect of calcitriol on the regulation of periodontal inflammation and the role of autophagy in this process. We found the defective autophagy and increased inflammation in the gingival tissues, especially in the epithelial cells of T2DM rats than non-diabetic rats. Additionally, calcitriol attenuated the gingival inflammatory responses in diabetic rats by promoting autophagy and LL-37 may be involved in this process.

Previous studies have demonstrated elevated inflammation in diabetic rats, which was confirmed by our study. The accumulation of ubiquitinated proteins in islet β cells (25, 26) and advanced glycation end product (AGE)-induced ROS (27, 28) cause the increasing inflammation. In the T2DM rats induced by HFD for 4-6 weeks combined with STZ injection (30-35 mg/kg), higher level of serum glucose and homeostatic model assessment-insulin resistance (HOMA-IR) were observed (29–31). However, the high-dose STZ (>50 mg/kg) injection can induce type 1 diabetes according the studies (32–34). In our experiment, we used HFD combined low-dose STZ (35 mg/kg) injection to establish T2DM rats, which is frequently used (24, 29–31). After HFD + STZ injection, the RBG of rats in our study ranged from 20.9 mmol/L to 28.2 mmol/L. The periodontitis model was established by ligation and *P. gingivalis* inoculation. Accumulation of plaque around the ligature and persistent infection with *P. gingivalis* can cause inflammatory infiltration, which leads to the destruction of periodontal tissues (35). Micro-CT and H&E staining revealed alveolar bone loss and inflammatory cell infiltration, which indicated a successful periodontitis model. IHC staining revealed the increased expression of NFκB-p65 in diabetic rats. NFκB-p65 is closely related to the expression of various pro-inflammatory mediators (36). Therefore, in this study, the experimental model established in rats with T2DM and periodontitis was successful and reliable.

This study revealed that the periodontitis condition induced a more severe inflammatory response in the gingival epithelium of diabetic rats than non-diabetic rats. Moreover, blocking of autophagy further aggravated the inflammation. Moderate autophagy plays a crucial role in general homeostasis by phagocytizing and degrading cargo (37). In our study, autophagy was interrupted with 3-MA. Consequently, we observed a decreased expression of autophagy flux, while the inflammatory response increased significantly. These data indicated that autophagy in the gingival epithelium of diabetic rats may decrease, which may weaken the degradation of inflammatory factors and in turn aggregate the periodontal inflammatory process.

In addition, this study revealed low expression of autophagy in the gingival epithelium of diabetic rats, which is rarely reported. The synthesis and processing of LC3 is increased during autophagy, making it a key readout of autophagy levels in cells (38). p62/SQSTM1 serves as an essential adaptor to identify and deliver specific organelles and protein aggregates to autophagosome for degradation (39). The detection of p62 turnover assay is reliable for autophagy flux assay (38). It has been reported that autophagy may play a protective role by clearing the aggregates of ubiquitinated proteins resulting from hyperglycemia in insulin-expressing β cells (25). Bachar- Wikstrom et al. (26) reported that large aggregates of autophagy and increased expression of LC3 was observed in pancreatic β cells of diabetic rats. High glucose was found to increase protein oxidation and trigger autophagy in MC3T3-E1 cells (40). In contrast, the expression of autophagic hallmarks (Beclin-1 and LC3), and numbers of autophagolysosomes was decreasing, and the expression of p62 was increasing in the diabetic hearts (41). The results of our study showed decreased expression of LC3-II/LC3-I and increased expression of p62 *via* IHC and western blot analysis, which indicated the insufficient autophagy in the gingival tissues of diabetic rats. However, the relevant mechanism should be explored further in the future.

Vitamin D can decrease the level of inflammation in diabetics (11, 12). However, the role of autophagy in the above process has not been reported. The present study revealed increasing autophagy and decreasing inflammation in rats gingival tissues treated with calcitriol, especially in the gingival epithelium. Meanwhile, calcitriol could attenuate the upregulation of NFκB-p65 and inflammatory response caused by autophagy inhibitor in our study. Similarly, vitamin D can protect against osteoarthritis, myocardial damage, and diabetic nephropathy by promoting autophagy (17–19). Vitamin D has been shown to increase the expression of LC3 and Beclin-1, suppress apoptosis, and alleviate insulitis in pancreatic β cells of diabetic mice (42). Therefore, we hypothesized that calcitriol may decrease periodontal inflammation by promoting autophagy and degrading inflammatory factors.

Our previous study indicated that LL-37 might promote autophagy of keratinocytes to reduce the quantities of live *P. gingivalis* (23). The results of this study revealed that expression of LL-37 was lower in the gingival epithelium of rats with T2DM compared with rats without T2DM, but LL-37 was promoted in gingival tissues with periodontitis both in non-diabetic and diabetic rats.

Turkoglu et al. (43) reported the increased levels of GCF LL-37 in patients with periodontitis. Furthermore, we found that calcitriol promoted LL-37 expression while increasing autophagy. Wang et al. (44) demonstrated that vitamin D played an important role in antimicrobial cutaneous immunity when identifying a vitamin D response element (VDRE) in the promoter region of the cathelicidin LL-37 gene. Soon thereafter, other groups confirmed that cathelicidin constituted a direct target of vitamin D in keratinocytes (45). Yuk et al. (46) indicated that calcitriol induced autophagy in human monocytes *via* LL-37, which activated transcription of Beclin-1 and Atg5. Nilsson et al. (47) reported that LL-37 antagonize pro-inflammatory cytokines produced by lipopolysaccharide- stimulated PDL cells, so we speculated the reducing of LL-37 after the application of 3-MA may be due to the consuming of LL-37 to neutralize excessive inflammatory factors in our study. In the present study, the decrease of LL-37 may be the specific manifestation in the gingival tissues with the severe periodontal inflammatory response induced by T2DM. Furthermore, calcitriol may promote the expression of LL-37 to take part in the inflammatory response of periodontitis in T2DM rats. However, the possible mechanism on the above process have not been clarified and need further study.

**Figure 7:**
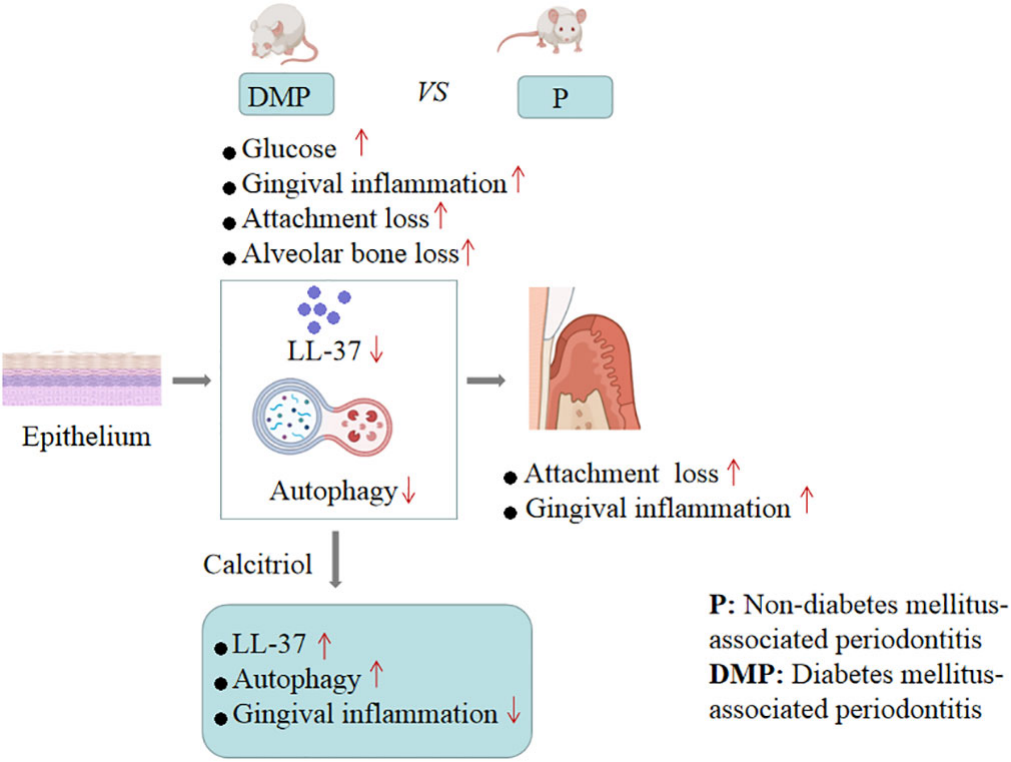
Mechanism schema diagram.

In conclusion, our findings provided evidence that calcitriol-enhanced autophagy positively attenuates periodontal inflammation and the decrease of LL-37 was rescued by calcitriol treatment in the gingival epithelial cells of T2DM rats (**Figure 7**). We will continue to study potential mechanisms to provide evidence for the application of calcitriol as an adjunctive treatment for T2DM-associated periodontitis.

## Data availability statement

The raw data supporting the conclusions of this article will be made available by the authors, without undue reservation.

## Ethics statement

The animal study was reviewed and approved by Animal Ethics Committee of China Medical University (KT2019040).

## Author contributions

XT, YW designed the study. YW performed the experiments with the help from XT, WX and FL. YW wrote the final manuscript. XT, MH and CM revised the manuscript. All authors contributed to the article and approved the submitted version.
